# Anomaly Detection IDS for Detecting DoS Attacks in IoT Networks Based on Machine Learning Algorithms

**DOI:** 10.3390/s24020713

**Published:** 2024-01-22

**Authors:** Esra Altulaihan, Mohammed Amin Almaiah, Ahmed Aljughaiman

**Affiliations:** 1 Department of Computer Networks and Communications, College of Computer Sciences and Information Technology, King Faisal University, Al-Ahsa 31982, Saudi Arabia; esra12989@gmail.com; 2 King Abdullah the II IT School, The University of Jordan, Amman 11942, Jordan; malmaiah@aut.edu.jo; 3 Faculty of Information Technology, Applied Science Private University, Amman 11931, Jordan; 4 Department of Computer Science, Aqaba University of Technology, Aqaba 11191, Jordan

**Keywords:** IoT network, DoS attacks, feature selection, classifier algorithms, machine learning, IDS

## Abstract

Widespread and ever-increasing cybersecurity attacks against Internet of Things (IoT) systems are causing a wide range of problems for individuals and organizations. The IoT is self-configuring and open, making it vulnerable to insider and outsider attacks. In the IoT, devices are designed to self-configure, enabling them to connect to networks autonomously without extensive manual configuration. By using various protocols, technologies, and automated processes, self-configuring IoT devices are able to seamlessly connect to networks, discover services, and adapt their configurations without requiring manual intervention or setup. Users’ security and privacy may be compromised by attackers seeking to obtain access to their personal information, create monetary losses, and spy on them. A Denial of Service (DoS) attack is one of the most devastating attacks against IoT systems because it prevents legitimate users from accessing services. A cyberattack of this type can significantly damage IoT services and smart environment applications in an IoT network. As a result, securing IoT systems has become an increasingly significant concern. Therefore, in this study, we propose an IDS defense mechanism to improve the security of IoT networks against DoS attacks using anomaly detection and machine learning (ML). Anomaly detection is used in the proposed IDS to continuously monitor network traffic for deviations from normal profiles. For that purpose, we used four types of supervised classifier algorithms, namely, Decision Tree (DT), Random Forest (RF), K Nearest Neighbor (kNN), and Support Vector Machine (SVM). In addition, we utilized two types of feature selection algorithms, the Correlation-based Feature Selection (CFS) algorithm and the Genetic Algorithm (GA) and compared their performances. We also utilized the IoTID20 dataset, one of the most recent for detecting anomalous activity in IoT networks, to train our model. The best performances were obtained with DT and RF classifiers when they were trained with features selected by GA. However, other metrics, such as training and testing times, showed that DT was superior.

## 1. Introduction

The IoT is present in many elements of our lives, including homes, cars, trains, streets, transportation, agriculture, and businesses. However, the increasing interconnectedness of these devices has raised concerns regarding the security and trustworthiness of IoT communications. As IoT systems become more complex and extensive, ensuring secure and reliable interactions among devices becomes a paramount requirement [1]. Trust and reputation management mechanisms play a pivotal role in establishing the reliability and integrity of IoT ecosystems. The rapid growth of such attacks is partly due to the proliferation of IoT technologies in areas such as smart grids, environmental monitoring, patient monitoring systems, smart manufacturing, and logistics [2]. The design of IoT devices entails several automated processes that enable them to self-configure. The devices discover and connect to networks autonomously using protocols like UPnP or mDNS, minimizing the need for manual intervention during setup. Plug-and-play capabilities enable these devices to establish connections instantly upon powering up or connecting, automatically identifying network protocols, and initiating operations without requiring explicit configuration. Additionally, these devices can be configured dynamically to obtain IP addresses and network settings, thereby ensuring adaptability within various environments. In addition to the configuration data they get from centralized servers and the cloud, they adjust the setting details based on specific deployment scenarios through auto-provisioning mechanisms. Furthermore, advanced IoT systems may incorporate machine learning algorithms for autonomously optimizing configurations over time, adapting to changing user behavior. The combination of automated processes allows IoT devices to configure themselves more efficiently and effectively, so user intervention is minimized, and their usability can be enhanced in a variety of network environments.

IoT systems are susceptible to a wide range of security attacks, and of those that prevent legitimate IoT network users from accessing their services, Denial-of-Service (DoS) is one of the most common. As a result, firms and organizations suffer huge monetary losses by violating Service Level Agreement (SLA) terms. Therefore, to ensure that systems, devices, and data are secured and protected effectively, robust techniques and mechanisms to counter attacks against IoT networks are needed [3]. In addition, DoS poses a danger to many IoT systems, including smart cities, healthcare applications, and agriculture systems. Attackers can exploit vulnerabilities in IoT devices, such as smart lights, door locks, and smart TVs, to launch DoS attacks against them [3]. One common attack type is distributed DoS (DDoS), one of which occurred on Friday, 21 October 2016, when many IoT devices were attacked. The attackers targeted IoT security vulnerabilities and hacked the IoT network, affecting access to customer data and disrupting services and applications. The companies sustained huge damage [4]. Many researchers in the literature have mentioned the urgency of the need to address the security of IoT systems and networks against DoS attacks, and some have proposed several DoS defense techniques to detect such attacks. Defenses against DoS attacks tend to include multiple verifications, network traffic filtration, and inspection of attacks. Most of these frameworks still have limitations, such as the use of traditional techniques and the lack of strict security requirements [3]. Very few studies have employed machine learning techniques in the Intrusion Detection System (IDS) to detect DoS attacks in IoT networks [3].

It has recently become possible to use the IoT paradigm to build smart environments that aim to maximize the comfort and efficiency of human life. A billion IoT smart nodes interconnect without human interaction through self-organized and heterogeneous communication networks. Various fields have been improved using IoT-based systems in recent years, including healthcare, agriculture, supply chains, education, transportation, and traffic monitoring. Of the 17 billion connected devices worldwide, 7 billion are IoT devices, a number that is expected to rise to more than 22 billion within the next few years [4]. Unfortunately, most of these connected devices offer inadequate security and privacy protection, triggering many security and privacy concerns in IoT networks. In turn, node heterogeneity has raised security concerns, one of the most challenging aspects of the IoT [5]. However, IoT device security cannot be achieved by employing security methods like IDS, access control, and authentication [6].

Therefore, researchers are concerned with securing these devices. The intrusion detection field has received heavy research attention worldwide in order to resolve this issue. IDSs designed specifically for IoT environments are crucial for mitigating IoT-related security attacks. An IDS is a security mechanism that works mostly at the network layer of an IoT system. For an IoT system to be fully effective, the IDS must be able to analyze packets of data at different layers of the IoT network with different protocol stacks, generate responses in real time and adapt to a variety of technologies based on the IoT environment [3]. Smart environments that rely on the IoT require IDSs with a high data volume, high performance and fast response time. As a result, conventional IDSs may not be completely suitable for IoT environments [7]. However, designing an efficient IDS for IoT devices remains a challenge for a number of reasons: detection systems for IoT environments must be extremely lightweight and use minimal storage space and computing power [8]. Also, for detection and classification, the number of features must be kept as simple as possible [8]. Using all features in the design of an IDS can introduce redundant and irrelevant features. To achieve effective IDS performance, feature optimization is necessary. Moreover, many of the existing datasets are outdated and may be inefficient at capturing the behavioral patterns of modern cyberattacks. However, little information is available about recent attacks or their patterns [7]. So, finding suitable datasets to train and test the detection system is challenging [7]. Therefore, this paper aims to:

- Propose an IDS defense mechanism based on anomaly detection and machine earning (ML) techniques in order to prevent hackers from successfully attacking IoT networks.
- Select the most relevant and important features using the CFS algorithm and GA and compare them.
- Determine the optimal ML classifier model for detecting and classifying DoS traffic in IoT networks by evaluating four types of supervised classifier algorithms, KNN, SVM, DT, and RF. Then, apply a confusion matrix to analyze the results.

This paper builds upon our previous study about the threats to IoT, in which we found that DoS attacks pose the most threat to IoT networks and that detection techniques are required to address this challenge [9].

## 2. Intrusion Detection System

**Figure 1:**
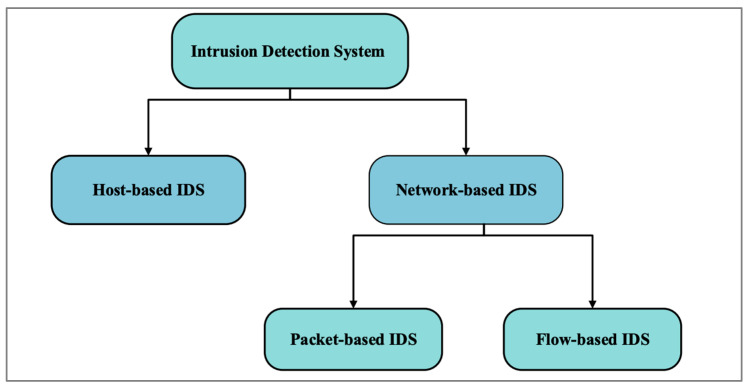
Types of Intrusion Detection Systems.

An IDS is a software or a service that monitors or identifies abnormal activity within a network or system. To predict anomalies in IoT networks, several ML methods are being implemented [10]. Figure 1 shows that IDSs are mainly classified into two types: host-based and network-based IDSs. A host-based IDS monitors and secures a single device or host based on the device’s information, such as system logs. In contrast, a network-based IDS measures the flow of data within a network by accessing and analyzing the data. A network-based IDS can be further divided into packet- and flow-based systems. Information from the network packet, such as the payload or header, is used for the packet-based IDS. As a result, it is often called the traditional IDS [11]. In contrast, the flow-based IDS analyzes and monitors anomalies within a network based on network flow characteristics, such as data rate and bytes. Thus, it is also known as network behavior analysis [11]. It is possible to classify malicious or anomalous network activity using supervised or unsupervised ML models.

## 3. Existing Work

Verma et al. [3] discussed the role of anomaly-based IDS in securing IoT against DoS attacks. A performance evaluation of seven ML classification algorithms is conducted, including RF, adaboost, gradient-boosted machines, extremely randomized trees, classification and regression trees, and multi-layer perceptron. To determine the optimal parameters of classifiers, they used random search algorithms. Various factors are taken into account when measuring classifier performance, including accuracy, specificity, sensitivity, false positive rate, and area under the receiver operating characteristic curve. The CIDDS-001, UNSW-NB15, and NSL-KDD datasets are used to benchmark all classifiers. To find significant differences among classifiers, Friedman and Nmenyi post-host tests are applied to the statistical analysis of performance measures. Furthermore, the authors evaluated all of the classifiers’ response times using the Raspberry Pi hardware device. Based on the performance results and statistical tests, they found that classification and regression trees, as well as extreme gradient-boosting classifiers, are optimal for building anomaly-based IDS that are tailored to the IoT environment.

Khatib et al. [6] presented ML solutions that detect and protect systems from abnormal states. Furthermore, several ML classifiers were used to analyze the effect of data oversampling on ML models. They also studied the binary and multiclass cases and compared the different techniques used after resampling and balancing the dataset using the SMOTE method. The results showed that when employing this kind of data to identify cyberattacks on IoT network traffic, Linear Discriminant Analysis (LDA), RF, DT, the approaches performed better than others since they were able to predict assaults with a higher degree of accuracy. Also, it was found that the DT, RF, and Nystrom-SVM techniques performed better in the binary case, which is the first time the same technique has been applied for detecting attacks in IoT network traffic. They noticed that when they trained their algorithms with balanced data, they were able to detect attacks more efficiently.

Khammassi et al. [12] presented a feature selection approach for IDS to produce a subset of features suitable for classifying the KDD99 and UNSW-NB15 datasets. There are three stages in the proposed approach: preprocessing, feature selection, and classification. During the preprocessing phase, redundant records are eliminated, the datasets are resampled, and attribute values are modified to make them compatible with Logistic Regression (LR) classifiers. The feature selection stage uses the Genetic Algorithm-Logistic Regression (GA-LR) wrapper, which involves an interaction between a GA-based feature search and an LR-based learning algorithm. Optimal subsets are selected by maximizing the accuracy of classification and minimizing features. As a result of the GA-LR wrapper, the best subset of features is used for classification. Three decision tree classifiers—C4.5, RF, and NBTree—are used to assess and compare the produced feature subsets against other current methods throughout the classification step. With only 18 features in the KDD99 dataset, the experimental results showed a 99.90% classification accuracy, a 99.81% detection rate (DR), and a 0.105% false alarm rate (FAR). In addition, the selected subset offers a good DR of 99.98% for the DoS category. For UNSW-NB15, the lowest FAR was 6.39%, and the classification accuracy was good in comparison to other approaches with a subset of 20 features. As a result of these findings, the UNSW-NB15 dataset is more complex than the KDD99 dataset. To improve the accuracy of classification for the new IDS benchmark, they suggest trying other approaches.

Mukherjee et al. [13] proposed to identify anomalies in smart devices and IoT systems. A supervised learning model was used to predict anomalies in historical data, which could be incorporated into real-world scenarios, preventing future anomalies and attacks. ML models are used to predict anomalies on 350 K datasets, and their performance is compared with the state of the art. Two different approaches are used based on the dataset analysis. Initially, the classification algorithms were applied to the entire dataset, and after excluding data points with binary values (0 and 1) in the feature “value”, the same classification algorithms were applied, and the results have been 99.4% accurate in the first case and 99.99% accurate in the second case.

Brun et al. [14] provided a methodology for network attacks against IoT gateways to be detected online. Using a set of metrics extracted from packet captures, the methodology predicts that there is a possibility of a network attack using a deep-learning approach. Based on empirical validation results on packet captures containing attacks, the Dense Random Neural Network (RNN) detects attacks correctly.

Tyagi et al. [15] developed an IDS based on extracted novel feature sets synthesizing the BoT-IoT dataset that can accurately and automatically distinguish benign and malicious traffic in real-time. An IoT-specific lightweight feature set consisting of seven lightweight features was developed instead of using existing feature reduction techniques such as principal component analysis (PCA), which can change the core meaning of variables. The study shows that fabricated seven features can be used to detect four types of attacks, such as distributed DoS (DDoS), DoS, reconnaissance, and information theft. Further, the study demonstrated the efficiency and applicability of supervised ML algorithms such as KNN, LR, SVM, multilayer perceptron (MLP), DTs, and RF in IoT security. A variety of performance metrics, including accuracy, precision, recall, F-Score, and receiver operating characteristics (ROC), are used to validate the performance of the proposed system. They found that DT and RF classifiers both performed nearly as well in terms of accuracy (99.9%), but other metrics like training and testing times indicated that RF was superior.

Thamilarasu et al. [16] proposed an intelligent IDS that detects anomalous behavior on insecure IoT networks by combining network virtualization with the Deep Learning (DL) algorithm. The IDS can detect attacks relating to the IoT, such as blackholes, opportunistic attacks, DDoS, sinkholes, and wormholes. An approach based on deep learning is used to detect attacks. In the proposed system, features are selected based on the information gained at each Deep Neural Network (DNN) layer. Based on the results of the proposed deep learning model, the true positive rate was 97%, and the average precision was 95% for all attacks. A total of five models have been developed for the detection of each attack type. In their experiments, they found that it is both practical and feasible to use DL algorithms for effective anomaly detection in the IoT environment, based on network simulations and testbeds.

Anthi et al. [17] proposed a framework that uses ML classifiers to identify network probing and simple DoS attacks (SYN flood, UDP flood). For DoS attack detection, the proposed system showed low precision (high false positive (FP)) and low recall (high false negative (FN)). Therefore, it does not deliver promising results for detecting attacks.

Ye et al. [18] combined the SVM classification algorithms with the simulation platforms of mininet and floodlight to construct the Software-Defined Networks (SDN) environment, and the 6-tuple characteristic values of the switch flow table were extracted. They also implemented deep packet analysis for detecting DDoS attacks in SDN by analyzing flow entries (such as source IP, destination IP, source port, destination port, and number of packets). Since the characteristic values are attack-dependent, they fail for other types of attacks besides DDoS attacks. Using the proposed IDS, the false alarm rate reaches 0%, which is virtually impossible in reality. Based on their experiments, the accuracy rate of their method is 95.24% with a small amount of data collection.

Kostas [19] proposed an anomaly detection network using ML methods. CICIDS2017 was used as a dataset since it is up-to-date and has a wide variety of attacks. An RF regression algorithm was used for feature selection on this dataset. A total of seven ML algorithms were used in the application step, all of which produced high performance. These are the following ML algorithms and success rates: Naive Bayes (86%), Quadratic Discriminant Analysis (QDA) (86%), RF (94%), ID3 (95%), AdaBoost (94%), MLP (83%), and KNN (97%).

Alsheikh et al. [20] proposed a framework that integrates RF algorithms for classification jobs and additive regression techniques for anomaly detection in medical wireless sensor networks (WSNs). The framework was tested on real medical datasets available from reliable sources and achieved both spatial and temporal anomaly detection. The research team found that ML algorithms and techniques can be instrumental in devising a framework for fault and anomaly detection in medical WSNs.

Hasan et al. [21] analyzed several ML models to predict attacks and anomalies on IoT systems. A number of ML algorithms were used, including LR, SVM, DT, RF, and artificial neural networks (ANN). In comparing performance, accuracy, precision, recall, and F1 score are used as evaluation metrics. A 99.4% test accuracy was achieved for DT, RF, and ANN. Even though these three techniques have similar accuracy, other metrics suggest that RF performs better.

Ramadan et al. [22] proposed a hybrid IDS system that detects IoT network attacks. Preprocessing and classification are the two stages of the proposed system. Data preprocessing is performed on the NSL-KDD dataset by encoding, scaling, and removing noise. Enhanced Shuffled Frog Leaping (ESFL) is then used to extract relevant features. A hybrid IDS system known as the Light Convolutional Neural Network with Gated Recurrent Neural Network (LCNN-GRNN) is used for classification. An anomaly class or a normal class depends on this classification. Based on the experimental results, the proposed system performed better than the existing methods.

Aversano et al. [1] proposed a DL-based anomaly detection method for IoT scenarios. Using a DNN architecture and 70 features, they detected anomalies in network traffic and identified the type of attack. Additionally, an autoencoder and a hyperparameter optimization analysis are used to reduce the number of features. To conduct experiments, they developed an integrated dataset based on public IoT traffic traces. In all the scenarios analyzed, the results show good performance. With all features included in the binary classification, the best accuracy is obtained at 99.89% in the top hyperparameter permutation. Furthermore, feature reduction leads to stable classifier performance (using a PCA and a 9-layer autoencoder, for example, to obtain accuracy greater than or equal to 99.2% when there are greater than 35 features). The 70 considered features in the multinomial classifier are too many, and fewer features are likely to provide better and more reliable results. However, when using a 9-layer autoencoder for feature reduction, the best accuracy is 98.9% obtained when the number of features is 60. When the number of features is between 35 and 60, this feature reduction approach ensures better performance. Finally, the optimized DNN architecture is evaluated in a noisy scenario that involves some of the features considered. It was also found that adding Gaussian noise in up to 40% of the features considered did not affect performance too much, especially for the binary case.

Lopez-Martin et al. [23] propose a new network intrusion detection method for IoT networks. Based on a conditional variational autoencoder, the proposed method integrates intrusion labels within the decoder layers. It provides better classification results than other familiar classifiers and is less complex than other unsupervised methods based on variational autoencoders. Furthermore, the method recovered missing features from the incomplete training datasets by performing feature reconstruction. The Network Intrusion Detection System (NID), which is part of network monitoring systems, and IoT networks can use both capabilities. This model performs exceptionally well for both tasks, performing better on the NSL-KDD test dataset, for example, than well-known algorithms: RF, linear SVM, multinomial logistic regression, and multilayer perceptions. The model creates a single model in a single training step, regardless of the labels associated with the training data. Classifiers based on variational autoencoders need to create as many models as there are distinct label values, each requiring a different training step. It takes a great deal of computational effort and resources to complete the training steps. Models can recover missing categorical features with three, 11, and 70 values, respectively, with 99%, 92%, and 71% accuracy. Multilabel classification and feature reconstruction problems are evaluated using extensive performance metrics. For predicted or reconstructed labels, they provided aggregated and one-versus-rest metrics, such as accuracy, F1, precision, and recall.

Yihunie et al. [24] analyzed anomaly-based IDS using ML techniques. A total of five ML classifiers were applied to the NSL-KDD dataset: stochastic gradient descent, RF, logistic regression, SVM, and sequential model. In spite of the fact that the NSL-KDD dataset does not accurately represent current network traffic, it is used in that research due to the lack of a publicly available dataset. Five different classification algorithms were tested with and without one-hot encoding. Based on the results, RF outperformed the other four classifiers. The RF classifier achieved near-perfect results. The RF model achieved the highest recall, indicating that a minimum number of false negatives were encountered.

Kim et al. [25] presented a GA to improve the detection model in network IDSs based on SVM. In addition, IDS should be able to handle misuses as well as novel attacks, and it should process audit data with minimal overhead on the computer system and IDS itself. The performance of SVMs has been shown to be better than that of traditional classification methods. It is true that SVM-based IDS can improve IDS performance in terms of detection rates and learning speed compared to conventional algorithms like neural networks (NNs), but there is still room for improvement. It is often the case that the overall performance of IDS is severely degraded as the number of features in the audit data increases. In order to overcome these problems, they employed the GA technique, which provides fast and excellent optimization for IDS, to determine the optimal detection model. On the KDD99 intrusion detection dataset, they demonstrated the feasibility of the proposed system. Finally, the paper method is not only capable of determining the optimal detection model but also minimizes the number of features that an SVM classifier should process and therefore maximizes detection rates.

Meng [26] conducted a broader and deeper experiment to compare the performance of NNs, SVM, and DT with the aim of demonstrating the practice and exploring the issues of using such types of approaches to detect network anomalies. Also discussed and analyzed are the effects of feature selection. Their experimental results indicate that ML approaches are capable of identifying anomalies with proper training, but the performance may vary depending on the algorithm. The wide use of ML schemes in real-life operational environments is hindered by fluctuations in capability, false alarm rates, and training data difficulties. In order to enhance detection performance, ML schemes should be applied in an appropriate manner.

Al-Janabi et al. [27] developed an anomaly-based IDS that uses artificial NLs to detect and classify attacks. Three main modes of operation are available for the developed system: detection mode (for distinguishing normal from abnormal actions), detection and classification mode (for further categorizing abnormal events into four types of attacks: DOS, PROB, U2R, or R2L), and detailed classification mode (for dividing abnormal events into 29 types of sub attacks). Additionally, anomaly intrusion detection parameters are applied to packet behavior. IDSs can learn a system’s behavior using several methods. To learn the behaviors of a system, the proposed IDS uses a back propagation ANN. KDD99 was used in their experiments, and the results were obtained to meet the objectives.

Shurman et al. [28] introduced a hybrid-based intrusion detection system (IDS) framework for IoT networks that can identify suspicious network traffic from any node. They ran datasets of IPs against the design to determine how it could identify strange packets on the network and block undesirable IPs before they become an initial DoS threat.

Mamatha et al. [29] proposed an intrusion acknowledgment system that is characterized by four essential stages: Aggregation of data, where groupings of framework packages are gathered. In the second stage, data preprocessing is performed, in which getting ready and test data are prepared and fundamental features are selected to distinguish between classes. During the period of data gathering, data is first compiled to make the central features, like those in the KDD Cup 99 dataset. The third stage involves preparing the classifier, where the model for the request is prepared. Lastly, the readied classifier is used to detect intrusions on the test data during attack affirmation. A channel-based fragment affirmation calculation, explicitly based on Flexible Mutual Information Feature Selection (FMIFS), has been proposed. The FMIFS calculations are adjusted according to Battiti’s figures in order to diminish the number of highlights. In order to reduce the plenitude of highlights, FMIFS proposes an adjustment as per Battiti’s calculations. In FMIFS, the excess parameter that is required in Mutual Information Feature Selection (MIFS) and Modified Mutual Information Feature Selection (MMIFS) is discarded. In spite of the fact that there is no set framework or rule for determining the best inspiration, this can be intriguing a little while later. To create an IDS, FMIFS and least-squares support vector machines (LSSVM) are combined. LSSVM works with correspondence objectives rather than distinction obstacles as an alternative to quadratic programming problems for solving social event issues. LSSVMIDS and FMIFS were studied using three well-understood impedance disclosure datasets: KDD Cup 99, NSL-KDD, and Kyoto 2006+. LSSVMIDS is computationally efficient when using the proposed feature selection algorithm.

Albulayhi et al. [30] developed and implemented a novel approach to feature selection and extraction for anomaly-based IDS. To select and extract relevant features, two entropy-based approaches are used: information gain (IG) and gain ratio (GR). After that, the best features are extracted using mathematical set theory: union and intersection. Four ML algorithms are used to train and test the model framework on the IoT intrusion dataset 2020 (IoTID20) and NSL-KDD datasets: bagging, multilayer perception, J48, and IBk. Based on the intersection and union, 13 and 28 relevant features (out of 86) were obtained for IoTID20 and 15 and 25 relevant features (out of 41) for NSL-KDD, respectively. In addition, they compared the proposed approach with other state-of-the-art studies. Based on the comparison, their proposed model scores a very high 99.98% classification accuracy.

Krishnan et al. [31] evaluated a number of supervised feature selection methods in order to predict malicious network traffic against IoT devices. Three different methods of feature selection were employed: sequential backward processing, sequential forward processing, and recursive feature elimination (RFE). Furthermore, for each selection method, three different logistic regression techniques were implemented. According to the study, all three methods of logistic regression (Support Vector Classifier (SVC), RF, and XGBoost) performed well with high accuracy. As a result, these techniques can be used in a supervised learning setting to predict an attack on IoT devices.

Qaddoura et al. [32] proposed a three-stage approach involving clustering with reduction, oversampling, and classification using a single-hidden layer feed-forward neural network (SLFN). A novel aspect of the paper is how the data reduction technique and the oversampling technique are combined to generate useful and balanced training data and the hybrid approach used for detecting intrusion activities. As part of the experiments, accuracy, precision, recall, and G-mean were evaluated. They were divided into four steps: assessing the impact of data reduction with clustering, evaluating the framework with basic classifiers, evaluating the effect of oversampling, and comparing it to basic classifiers. It is found that SLFN classifications and SVM with Synthetic Minority Oversampling Technique (SVM-SMOTE) with 0.9 ratio and k value 3 for the k-means++ clustering technique produce better results than other classification techniques and other values.

Choudhary et al. [33] propose an IDS based on deep learning using SVM and DNN. Since SVMs are binary classifiers, only two routes were classified at a time for the appropriate route to be shown. Cosine similarity measures and deployment models have been introduced to understand route similarity. Measurements of precision, recall, F-measure, and accuracy have been performed to assess the proposed work. A comparison of the proposed architecture with other state-of-the-art techniques has been conducted to determine its effectiveness. Compared with other approaches, precision, recall, F-measure, and accuracy improved by 13%, 74%, 71%, and 76%, respectively.

Mohan Sai et al. [34] utilized a Raspberry Pi to implement a lightweight intrusion detection technique using a ML approach. Their method of classifying attack traffic and normal traffic was based on an SVM. To reduce the number of features in a dataset, a CFS algorithm is used. The dataset used was UNSW-NB 15, and the features were reduced from 44 to 3. These three features were used to train the model. The CFS model was compared to the non-CFS model using a laptop since it was impossible to train the model using all 44 instances on a Raspberry Pi, resulting in system failure. In the evaluation, the DoS attack instances are extracted from UNSWNB 15 and evaluated using the WEKA application. By reducing the number of features in the system, the CFS algorithm makes the system lightweight by enhancing detection accuracy. As a result of their experiments, they have found that their approach for detecting a DoS attack has a satisfactory detection rate and accuracy.

Naung Soe et al. [8] also developed an IoT cyber-attack detection system using ML technology. For the feature selection, they employed a lightweight and efficient feature selection algorithm, CFS, and a well-known DT version, the J48 algorithm, to carry out the classification. The system was implemented on a Raspberry Pi, and its performance was evaluated using UNSW-NBI5. During the experiment, the system was able to handle all the instances in this dataset and had a much faster training speed while retaining almost no degradation in detection accuracy. Moreover, if the step of feature selection was not taken, the system would only be able to handle 80% of the instances. The analysis of the above works is listed in Table 1.

In Table 2, the related literature is compared to our proposed system based on the following characteristics:

- **Dataset:** name of the dataset used to train the system.
- **Feature selection approach:** approach or algorithm used to select the best feature for detecting and classifying the attacks.
- **Performance metrics:** the metrics used to evaluate the performance of the system.
- **Average detection rate or accuracy:** the average or best detection or accuracy rate the system achieved.

**Table 2:** Experimental comparison of related work.

Author	Dataset	Performance Metrics	Feature Selection Approach	Average Detection Rate or Accuracy
Aversano et al. [1]	Integrated dataset based on public IoT traffic traces	Accuracy and F-measure	Autoencoder and a hyperparameter optimization analysis	The best accuracy is obtained (0:9989 for the top hyperparameter permutation).
Verma et al. [3]	CIDDS-001, UNSW-NB15, and NSL-KDD	Accuracy, specificity, sensitivity, false positive rate, and area under the receiver operating characteristic curve	Random search algorithms	96.74% By using CART
Khatib et al. [6]	UNSW-NB15	Accuracy, recall, F1 score, and ROC AUC curve	Not mentioned	95% accuracy with DT, RF, and Nystrom-SVM
Naung Soe et al. [8]	UNSW-NB 15	Processing time and detection accuracy	CFS	Not mentioned
Khammassi et al. [12]	KDD99 dataset and the UNSW-NB15 dataset	Accuracy, Recall, DR, FAR	Wrapper, GA, and LR	With a subset of only 18 features, the KDD99 dataset showed 99.90% accuracy of classification, 99.81% DR, and 0.105% FAR.
Mukherjee et al. [13]	From the ML data repository Kaggle, which was provided by Xavier.	Accuracy	Not mentioned	99.4% accuracy in the first case and 99.99% accuracy in the second case
Brun et al. [14]	Own a simulated dataset	Time series of the difference between the numbers of initiated and established TCP connexions per time slot, attack probability predicted by the dense RNN	Not mentioned	Time-series of the difference between the numbers of initiated and established TCP connections per time slot (10 s)
Tyagi et al. [15]	BoT-IoT dataset	Accuracy, precision, recall, F-Score, and ROC	Manual	There is no difference in accuracy between the DT:99.9% and RF: 99.9% classifiers.
Thamilarasu et al. [16]	The dataset consists of 5 million network transactions.	Precision, recall, and F1 Score	The selected features are transmission rate, reception rate, transmission-to-reception ratio, duration, transmission mode, source-IP, destination-IP, and data-value information.	Avg. Precision = 96.88%, Avg. Recall = 98.02%, F1 Score = 0.974
Anthi et al. [17]	Own a simulated dataset	Precision, recall, and F-measure	-	Precision: 97.7%; recall: 97.7%; F-measure: 97.7%
Ye et al. [18]	Own a simulated dataset	Detection accuracy rate, false alarm rate	Source IP, Destination IP, Source Port, Destination Port, and Number of Packets	Average detection accuracy rate: 95.24%; average false alarm rate: 1.26%
Kostas [19]	CICIDS2017	Accuracy, precision, F-measure, and recall	Random Forest Regressor algorithm	NB: 86%; QDA: 86%; RF: 94%; ID3: 95%; AdaBoost: 94%; MLP: 83%; KNN: 97%
Alsheikh et al. [20]	Real medical dataset	ROC curve, absolute error, and run-times	Not mentioned	Not mentioned
Hasan et al. [21]	An open-source dataset was collected from Kaggle	Accuracy, precision, recall, F1 score, and area under the receiver operating characteristic curve	No ML approach has been used for feature selection	99.4% test accuracy for DT, RF and ANN
Ramadan et al. [22]	NLS-KDD	False Positive Rate (FPR), accuracy, True Positive Rate (TPR), False Negative Rate (FNR)	The feature selection process is done by using the Enhanced Shuffled Frog Leaping (ESFL) algorithm.	Accuracy of 90.25% in attack detection compared to existing methods
Lopez-Martin et al. [23]	NSL-KDD	Accuracy, F1 score, precision, and recall	Latent multivariate probability distribution	Accuracy of 99%, 92%, and 71%, respectively
Yihunie et al. [24]	NSL-KDD	Precision, recall, F1 score, ROC curve, and accuracy	One-hot encoding	Accuracy: 99%, precision: 0.9992, recall: 0.9969, F1 score: 0.9980
Kim et al. [25]	KDD99	Detection rates	GA	Detection rates are more than 99%.
Meng [26]	KDD99	Accuracy and detection rate	Manual	All three algorithms achieve a very high detection rate, more than 94%.
Al-Janabi et al. [27]	KDD99	Detection Rate (DR) and False Positive Rate (FP)	Manual	Not mentioned
Shurman et al. [28]	Dataset of IPs	Detection time, accuracy	Not mentioned	Not mentioned
Mamatha et al. [29]	KDD99, NSL-KDD, and Kyoto 2006+	Accuracy, affirmation rate, false positive rate, and F-measure	Flexible Mutual Information Feature Selection (FMIFS)	100%
Albulayhi et al. [30]	IoTID20 and NSL-KDD	Accuracy, recall, precision, F1 Score, and ROC	Information gain (IG) and gain ratio (GR)	99.98% classification accuracy
Krishnan et al. [31]	IoTID20	Accuracy, F1 score, recall, RMSE	Sequential Backward Processing, Sequential Forward Processing, and Recursive Feature Elimination (RFE)	Best accuracy: 99.79%
Qaddoura et al. [32]	IoTID20	Accuracy, precision, recall and G-mean	Not Applied (all features used)	98.42%
Choudhary et al. [33]	Own a simulated dataset	Accuracy, recall, precision, and F-measure	Not mentioned	Precision: 98.12%, recall: 98.04%, F-measure: 94.88%, accuracy: 98.68%
Mohan Sai et al. [34]	UNSW-NB 15	F-measure, recall, precision FPR, TPR, TNR, True Negative Rate (TNR), and accuracy	CFS	Accuracy: 98%

### Discussion of Related Work

Based on the studies reviewed in the previous sections, we found the following:

- Most papers targeted the IoT network environment when designing their systems (e.g., [6,8,13,18,22,23,28,33]). Few papers targeted other environments, such as wireless medical sensor networks (e.g., [20]).
- Some papers used only one ML classifier for detection (e.g., [8,34], while others used multiple algorithms and compared their performance to determine the best one (e.g., [26]).
- In terms of the types of attacks identified, most of the papers trained their systems on multiple attacks, while only a few papers focused on specific attacks like DoS (e.g., [3,8]) and DDoS (e.g., [18]) attacks.
- As for the datasets used to train the system, most papers (e.g., [3,8,24]) used the UNSW-NB15, NLS-KDD, and KDD99, which are considered good but outdated datasets, while other papers built their own datasets ([14,17,18]). In some papers, however, the IoTID20 and Bot-IoT datasets, which collect data in real time, were used (e.g., [15,32,33]).
- In most of the studies, accuracy, recall, F1 score, and precision were used as metrics for evaluating system performance (e.g., [15,16,19,23]).
- Some of the papers that performed feature selection steps to select the important and relevant features [12] combined GA and LA, ref. [19] used RF, ref. [25] used GA, ref. [29] used the FMIFS algorithm, and ref. [8,34] used the CFS algorithm.
- Additionally, most of the papers in the review achieved high levels of accuracy in detecting their targeted attacks.

## 4. Methodology

To detect anomalies in IoT devices, ML classification algorithms will be used to build an IDS. This IDS will continuously monitor network traffic for any deviation from normal network profiles based on anomaly detection. There are three types of IDSs that can be used: signatures, anomalies, and specifications. Due to its ability to detect new attacks, an anomaly-based IDS is preferred over a signature- or specification-based IDS. However, it comes with a high false alarm rate. The effectiveness of an anomaly-based IDS depends on the quality of its detection engine (model or classifier) [3]. An anomaly-based IDS continuously monitors network traffic for deviations from the normal profile. As soon as a deviation exceeds the threshold, an alarm signals the detection of a DoS attack.

**Figure 2:**
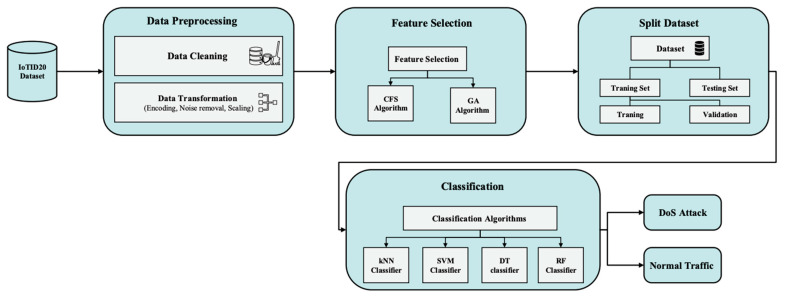
System workflow.

This study will have several key stages, as shown in Figure 2. To train the system, we will use the IoTID20 dataset, which contains recent types of attacks on IoT networks. We will do some preprocessing on this dataset to improve its quality and prepare it for feature selection and training. Data preprocessing includes cleaning and transformation of the data. Data cleaning involves removing null values and their entries. Whenever there are missing values, the ML algorithm has a difficult time handling them, and the model may produce an incorrect prediction. In addition, the data transformation will include encoding, noise removal, and scaling. In the initial dataset, there are many columns with multiple labels. A label can be defined in the form of a number or a word. Encoding refers to converting labels from a human-readable to a machine-readable format. With the help of filters, noise in the form of irrelevant features is removed by the noise removal process. Scaling is used to scale large amounts of numerical data based on distance values. Following these methods, the final features will be selected from these prepossessed features.

The scaled data will be used as input in the feature selection phase to select the most relevant and important features using a CFS algorithm and GA. After that, we will make a comparison between the feature selection algorithms and select the most appropriate one. After the features are selected, the dataset will be divided into two subsets: the training subset and the test subset. By selecting the right testing and training data, classification accuracy will be improved. The training data is the set of instances trained on the model, while the test data is used to determine the model’s ability or execution. Classification determines whether the information belongs to a normal class or a DoS attack. In order to achieve the best classification approach, a variety of algorithms, such as KNN, SVM, DT, and RF, will be compared. Finally, we will validate our proposed scheme using a confusion matrix.

### 4.1. Dataset

There are many datasets that can be used to train the system to detect DoS attacks. The dataset must contain real-time network traffic. It is essential that the dataset be versatile and extensive. In addition, the dataset should cover a variety of attack vectors and include the most recent DoS attacks [35]. According to the literature analysis in Section 2, the most commonly used datasets are UNSW-NB15, KDD 99, NSL-KDD, IoTID20, and Bot-IoT. We compared these available datasets in Table 3 to select the dataset with the highest quality to train our system.

According to this compression, the IoTID20 was selected in this study to train the IDS to detect DoS attacks, as it contains the most recent traffic attacks collected in real-time and scans simulate attacks on IoT networks.

#### IoTID20 Dataset

In the IoTID20 dataset, there are different types of IoT attacks (e.g., DDoS, DoS, Mirai, and ARP Spoofing) as well as normal (benign) traffic. IoTID20 is a dataset collected from smart home IoT ecosystems. Smart homes typically incorporate a variety of interconnected components, including artificial intelligence speakers (SKTNGU), Wi-Fi cameras (EZVIZ), laptops, smartphones, tablets, and wireless access points (Wi-Fi). In this dataset, the cameras and artificial intelligence speakers were the IoT victim equipment, and the other equipment was the attacking equipment. Using Nmap tools, attacks such as DoS, scanning, and man in the middle were simulated. Mirai botnet attacks were generated separately on a laptop, and then changed to simulate their origin on IoT devices [36]. Using the CICFlowMeter, the IoTID20 dataset created CSV files from these packet files. Based on the IP addresses, the CSV files were labeled according to their anomalous behavior and types of attacks. Table 4 and Table 5 show the distribution of the dataset. The dataset contains 86 features.

### 4.2. Feature Selection Algorithms

In order to improve the detection accuracy and training speed of our system, we needed to use a feature selection algorithm. Feature selection involves eliminating irrelevant and redundant features and selecting those that are most pertinent and relevant. For the feature selection phase, we decided to use two feature selection algorithms and compress between them, which are GA and CFS.

#### 4.2.1. Genetic Algorithm

**Figure 3:**
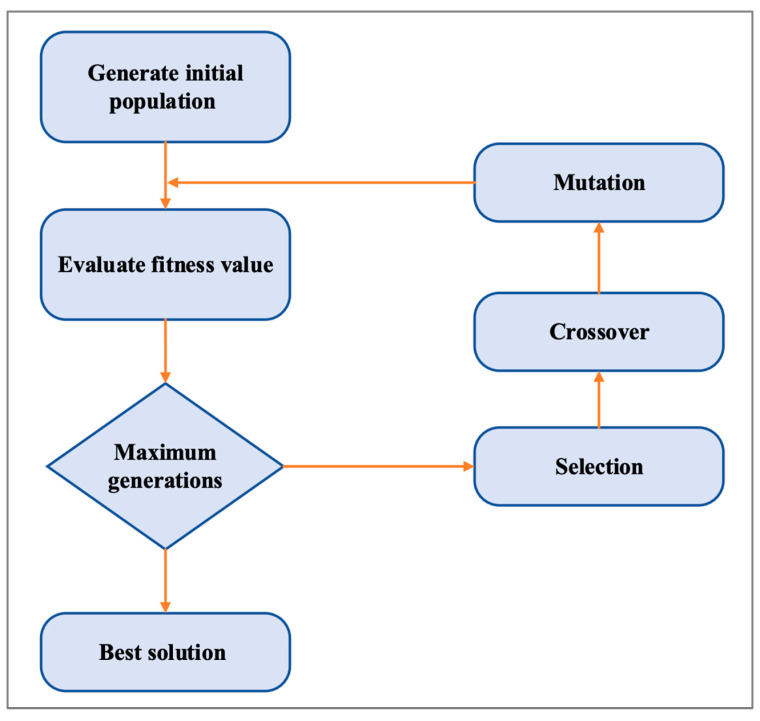
Genetic algorithm process.

The first algorithm is GA, which is a natural selection-based optimization technique. In GA, a set of optimal values is determined based on evolution. The first step in feature selection is to create a population from subsets of the possible features. Based on this population, subsets are evaluated using a predictive model. In order to determine which subsets of the population will continue into the next generation, each member of the population is considered in a tournament. The next generation is made up of the tournament winners with some cross-over (the winning feature sets are updated with features from other winners) and mutation (some features are introduced or removed randomly) [37]. Figure 3 shows how the algorithm works. As a result of the algorithm running for a set number of generations (iterations), the optimal member of the population becomes the selected feature.

#### 4.2.2. Correlation-Based Feature Selection

The CFS algorithm is a filter approach used to evaluate the correlation between outputs and input features [38]. According to empirical evidence, irrelevant or redundant information, which can increase computation time and result in poorer detection accuracy, should also be eliminated [8]. Redundant features are those that are highly correlated with other features. According to the CFS algorithm, a good feature subset consists of features that are highly correlated with the class and uncorrelated with each other [8]. The CFS algorithm supports most attribute types, including binary, date, nominal, empty, or unary value attributes [39]. As shown in Table 6, both algorithms have advantages and disadvantages.

### 4.3. Classification Algorithms

There are two types of ML algorithms: supervised and unsupervised. In supervised algorithms, predefined (classified) objects are used to predict the object class. Unsupervised algorithms, in contrast, find the natural grouping of unlabeled objects [40]. In order to obtain the best performance in our IDS, we will use and compare four types of supervised learning algorithms for classification.

#### 4.3.1. Decision Tree

**Figure 4:**
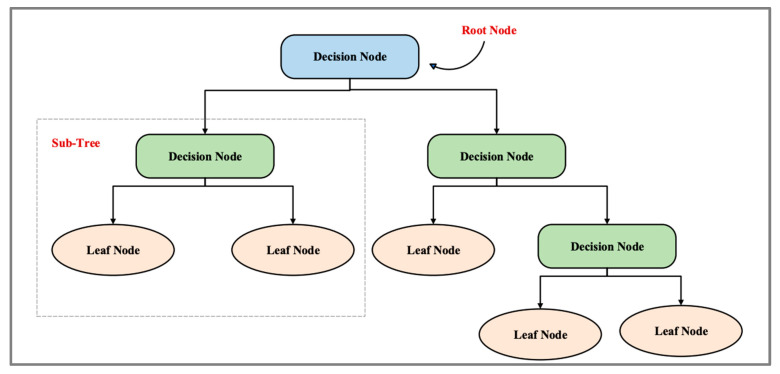
DT structure.

The first classification algorithm chosen to determine its performance in classifying the DoS attack is DT. This technique is used for solving both classification and regression problems, but generally, it is used for classification problems [41]. This classifier is tree-structured, where the internal nodes represent the dataset’s features, the branches represent the decision rules, and the leaves represent the outcome [42]. DT has two nodes: the decision node and the leaf node. Leaf nodes are the outputs of decision nodes and do not contain any further branches, whereas decision nodes are used to make decisions and have multiple branches. Dataset features are used to make decisions or perform tests. This is a way of getting all the possible solutions based on given conditions for a problem or decision. DT is similar to trees in that it begins with a root node, which expands into branches and creates an overall tree-like structure. In a decision tree, a question is asked, and based on the answer (yes or no), a subtree is created. Figure 4 explains the general structure of DT.

#### 4.3.2. Random Forest

The second classifier algorithm that was selected is RF. Using the RF classifier, a subset of the training set is randomly selected to create a set of decision trees. This method basically involves building multiple DTs from a randomly selected subset of the training set and then combining the votes from each tree to make a final prediction [43]. Taking data input, a classifier model assigns it to one of several categories. As an example, a classifier can be used to predict whether an image is that of a dog or cat, given a set of images containing images of dogs and cats. Basically, an RF algorithm creates multiple DTs, each based on a random subset of data. A DT is a type of algorithm that determines which category data inputs fall into based on the data inputs. By creating multiple decision trees and averaging their results, RFs go one step further. In this way, overfitting is reduced, which occurs when the algorithm only works well with the training data and not with the new data [43].

**Figure 5:**
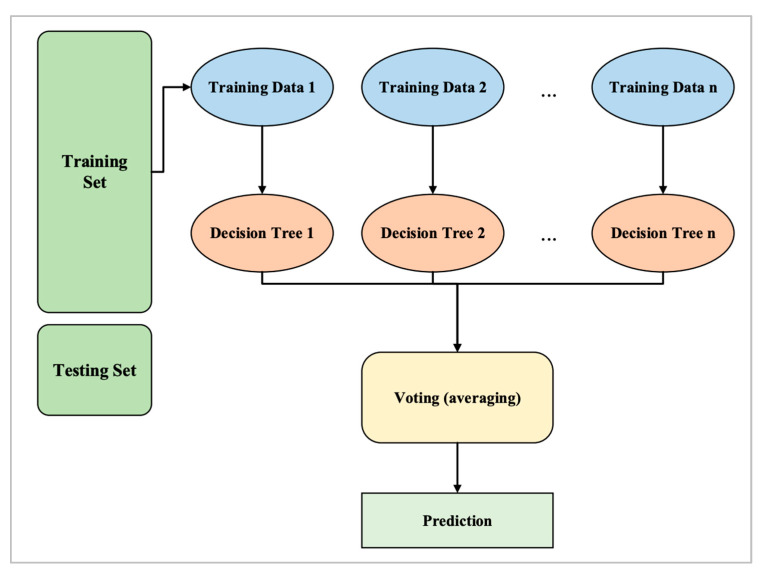
Structure of RF.

It is possible to think of the RF as an ensemble of several DTs. A final outcome is created by aggregating the predictions of multiple decision trees (majority voting) and averaging them. As a result, the RF model generalizes better to a larger population. Furthermore, the model becomes less prone to overfitting or high variance [44]. The RF algorithm steps are shown in Figure 5.

#### 4.3.3. Support Vector Machine

The third classifier was SVM. SVM is generally considered a classification approach, but it can also be used to solve regression problems. In addition to handling continuous variables, it can also handle categorical variables easily [45]. To separate different classes, SVM constructs a hyperplane in multidimensional space. Iterative SVM generates optimal hyperplanes, which minimizes errors. SVM is based on finding a maximum marginal hyperplane (MMH) to divide the dataset into classes [45].

The main objective is to separate the dataset as effectively as possible [46]. The distance between two points is known as the margin in SVM. Using the dataset given, the objective is to find a hyperplane with the greatest margin between support vectors. SVM undertakes the next steps to determine the MMH:

1. Create hyperplanes that segregate classes in the best way.
2. Select the right hyperplane that should have the maximum separation from the nearest data point.

#### 4.3.4. k-Nearest Neighbors

The kNN algorithm is a supervised ML algorithm that learns from labeled input data and uses that knowledge to produce accurate outputs with unlabeled data. kNNs are used to predict test data sets based on the characteristics (labeled data) of training data. Predictions are made by calculating the distance between test data and training data, assuming that the data points have similar characteristics [47]. The kNN algorithm is similar to a voting system, in which the majority class label determines the class label of a new data point among its nearest k (k is an integer) neighbors. Suppose you live in a small village with a few hundred residents, and you must decide which political party to support [47]. For this, you might ask your nearest neighbors which political party they support. Generally, if most of your nearest neighbor’s support party A, you will vote for it as well [47]. Similar to kNN, a new data point’s class label is determined by its k nearest neighbors by determining the majority class label.

### 4.4. Evaluation Metrics

Our proposed system was evaluated on accuracy, precision, recall, and F1 score. The evaluation metrics such as accuracy, precision, recall, and the F1 score are computed using these four parameters: true positive (TP), false negative (FN), false positive (FP), and true negative (TN).

#### 4.4.1. Confusion Matrix

A confusion matrix is a table used to describe the performance of a classifier within the context of a set of observations known to have actual (monitored) values. A change in a box refers to a skill in a lesson, and a change in a column refers to a skill in the intended course (or vice versa). A confusion matrix consists of the following terms:

- TP: Both the actual and predicted values are positive.
- TN: Both the actual and predicted values are negative.
- FP: The actual value is negative, but the model predicted it to be positive.
- FN: The actual value is positive, but the model predicted it to be negative.

We calculated several measures from the confusion matrix to quantify and compare the quality of the models, including the following:

##### Accuracy

The accuracy indicates that the flow manifests are accurately categorized around the entire traffic trace. It is the proportion of correctly classified cases above all N cases. The accuracy formula is as follows:

$$\mathrm{Accuracy}=\frac{TP+TN}{TP+TN+FP+FN}\times 100$$

##### Precision

Precision refers to how many intrusions an IDS predicts. A higher P indicates a lower alarm. The percentage of positive classifications made in the right direction. Following is the formula for calculating precision:

$$\mathrm{Precision}=\frac{TP}{TP+FP}$$

##### Recall

Recall measures how much of an intrusion was expected versus how many intrusions actually occurred, so a high R-value is needed. This is the percentage of positive examples that have been classified correctly. Following is the formula for calculating recall:

$$\mathrm{Recall}=\frac{TP}{TP+FN}$$

##### F1 Score

The F1 score provides an overall score by combining precision and recall. The model correctly identifies threats when the number of false alarms is minimal. The F1 score is good when there are false positives and false negatives. Below is the formula for the F1 score.

$$\mathrm{F1 score}=2\times \frac{Precision\times Recall\;}{Precision\times Recall}$$

## 5. Experiment

The software has been developed on a machine operated with 64-bit Windows 11 Home and equipped with an 11th Gen Intel^®^ i7-11370H four-core CPU having a 3.30 GHz clock speed and 16 GB of main memory. In order to implement the system, we first set up the development environment. PyCharm was chosen as our main IDE to code, run, and test the system. Additionally, the Skit-Learn platform was used to implement and test machine learning algorithms for classification. Our first step was to download Python 3.9.13 and PyCharm Professional in order to work with Jupyter Notebooks. We also imported and used several Python modules for the system’s functionality. The experiment process breaks down into multiple steps, including loading the data, data preprocessing, feature selection, splitting the dataset into training and testing sets, classification algorithms, and evaluating performance.

### 5.1. Load the Data

For training our system to detect DoS attacks, we chose the IoTID20 dataset due to its richness of features, as shown in Table 4. First, we downloaded the dataset from site [48] and saved it to our local computer. Since we only need to work with DoS and normal data from the dataset, in the next step, we filtered our dataset to retain only the data from the DoS and normal categories.

### 5.2. Data Preprocessing

For the data preprocessing, we performed a number of manipulations to enhance the data for training purposes including cleaning, feature removal, and encoding.

#### 5.2.1. Cleaning

In data cleaning, null values should be removed along with their entries. It is difficult for ML algorithms to handle missing values, so the model may produce incorrect predictions when missing values are present [4]. Because of that, we checked for null values with the help of the .isnull() function. The output of our dataset was 0 for all fields, and no null files were found.

#### 5.2.2. Features Removal

For the model to perform well, it was important to remove flow identifiers such as source and destination IP addresses, flow IDs, and timestamps. It will fail to generalize well if the model is trained using these features since attackers may use different IP addresses and times to launch attacks. So, we dropped them from the dataset. In addition to this, label and subcategory features are also deleted as they are not useful for achieving high accuracy and are difficult to handle in the next phases. The deleted features are mentioned in Table 7, and the remaining 80 features are in our dataset.

#### 5.2.3. Encoding

Labels are encoded to convert them from a human-readable to a machine-readable format. In this phase, we needed to convert every string to a numerical value. By using the .map() function, we mapped each “DoS” string to 1 and each “Normal” string to the numerical value 0, as shown in Table 8.

We observed that there is no need for scaling or noise removal steps in our case. The dataset has more integers and no outliers, so scaling is not useful. Additionally, noise removal is not required since the dataset has no noise.

### 5.3. Feature Selection

As we mentioned in Section 4.2 for the feature section, we decided to use two feature selection algorithms and compress between them. These algorithms are GA and CFS.

In GA, a population of potential feature subsets is iteratively evolved toward an optimal solution. Its functions are as follows: An initial set of features is generated in several subsets. Following that, individuals (feature subsets) are selected based on an evaluation metric that determines their fitness. An individual’s features are then enhanced by crossing with those of another individual, similar to genetic crossover. To maintain diversity, some individuals undergo random mutations. Individual fitness levels are reevaluated. Eventually, the process terminates after a certain number of generations (e.g., the process iterates until a termination criterion has been met) [37]. There are several variables in GA to consider. Population Size: the number of potential feature subsets in each generation. A crossover rate is the probability that two individuals will cross over. Mutation Rate: the likelihood of an individual becoming mutated [37].

In CFS, features are analyzed by their relationship to the target variable and their interrelationships. As a result, it functions as follows: A feature is evaluated independently based on its correlation with the target variable. Then, the evaluation of subsets of features involves considering both their correlation with the target variable and their redundancy. A merit function determines the overall worth of a feature subset by combining the evaluations of each individual feature and each subset [38]. An important variable in CFS is the Correlation Threshold, which defines the threshold at which individual features are correlated with the target variable. Subset Evaluation Metric, which is a measure used for evaluating the worth of feature subsets, is typically a combination of correlations and intercorrelations [39].

Both GA and CFS were influenced by adjusting these variables in the context of feature selection.

When we ran the GA as a feature selection algorithm with our dataset, the algorithm selected 13 out of 80 features as relevant and most important features to train our system. These features and their descriptions are listed in Table 9.

The CFS algorithm, on the other hand, arranges the features from the most important to the least important when applied to our dataset. However, we used only the first 13 important features selected by the CFS algorithm to make a fair comparison with the GA algorithm. These 13 features selected by the CFS algorithm are listed in Table 10.

There is only one feature that both algorithms selected in common, which is the Flow_IAT_Mean. The other 12 features are different for each.

### 5.4. Split the Dataset to Training and Testing Sets

The dataset must be divided into two subsets: a training subset and a testing subset. It is possible to improve classification accuracy by selecting the right testing and training data [49]. In training data, the model is trained on instances, while testing data determines the model’s ability to execute. We have split the dataset into 33% for testing and 67% for training. As a result, we had 66,640 data points in the training set and 32,824 in the testing set.

### 5.5. Classification Algorithms

Afterward, we worked with classification algorithms to determine if a packet belongs to the normal category or if it is a DoS attack. The best classification algorithm was determined by comparing a variety of algorithms, including DT, RF, KNN, and SVM. They were evaluated based on evaluation metrics, which include accuracy, precision, recall, and F1 score. Each of these algorithms will be trained in three ways: first, using all features (without applying any feature selection techniques), and then using features selected by GA. Finally, we will train it with features selected by CFS.

### 5.6. Evaluate the Performance

Our evaluation of the performance was based on the confusion matrix. Which will help us to compare classifier algorithms with and without feature selection algorithms (GA and CFS). In addition, to evaluate the classifier algorithms, we chose to use accuracy, precision, recall, and the F1 score.

## 6. Results and Analysis

This section presents and analyzes the experiment results, including the training and testing times, and evaluates the performance metrics.

### 6.1. Training and Testing Time

Table 11 presents the training and testing time with 12 models comprising four classifiers (DT, RF, SVM, and kNN) across three cases (trained with all features in the data set, trained with features selected by the GA, and trained with features selected by the CFS algorithm).

**Figure 6:**
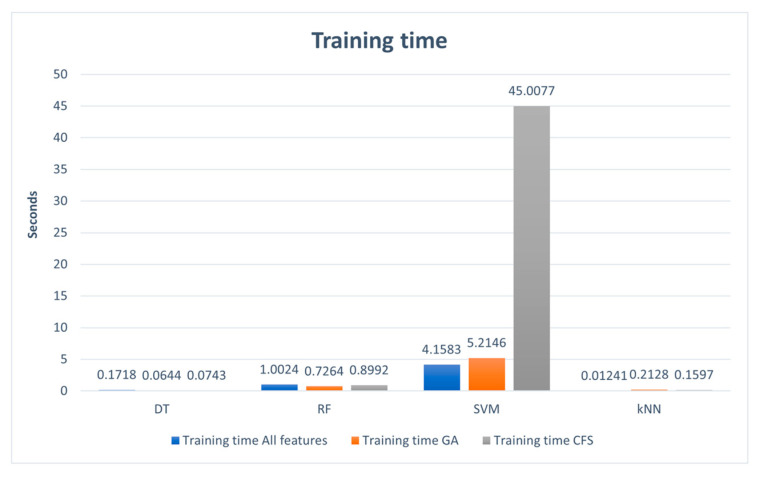
Training time.

In Figure 6, the training time is compared across four classifiers (DT, SVM, RF, and kNN) and three features’ cases (trained with all features, trained with GA-selected features, and trained with CFS-selected features). The kNN and DT algorithms were faster in the training process, with less than one minute in all cases. This is because one of the advantages of the DT is that it is very fast, and the kNN is known as a lazy algorithm because it does not use the training data to generalize, which makes it faster. The faster training time was achieved when training KNN with all features (0.1241 s). On the other hand, the slowest training time was obtained when training the SVM with features selected by CFS algorithms (45.0077 s). Because SVM training complexity varies greatly with the size of the dataset, it is not suitable for the classification of large datasets that have strong correlations between features, which is part of how CFS algorithms work and select the features.

**Figure 7:**
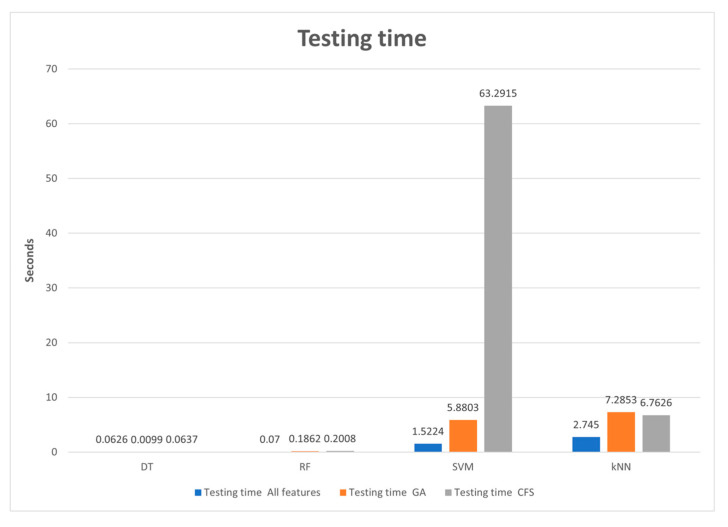
Testing time.

Figure 7 depicts a comparison of testing times for the four classifiers (DT, SVM, RF, and kNN) and the three feature scenarios (trained with all features, trained with GA chosen features, and trained with CFS-selected features). When GA chose the features for DT, the testing time was reduced to 0.0099 s. The slowest training time was obtained when the SVM was trained with features picked by CFS methods (63,2915 s). DT and RF algorithms provide the fastest testing times. Even though KNN took less training time, it took a longer testing time than DT and RF. This happened because, during prediction time, distances to the original training data are calculated (to determine which one is its closest neighbor), while training time does not need to calculate very expensive distances. It is mostly about moving from .fit() to .predict(). Trying to predict the train schedule takes more time.

From these results, we can conclude that the best classifier, which provided the optimal time for both training and testing, is the DT algorithm.

### 6.2. Evaluate the Performance

**Figure 8:**
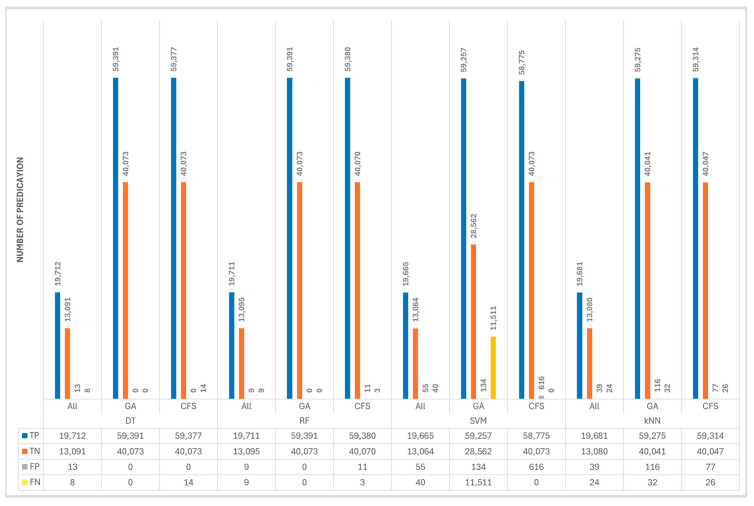
Evaluation of the performances.

Figure 8 illustrates the results of confusion matrix terms, including TP, TN, FP, and FN. The true statement indicates that the values were accurately predicted, whereas the false statement indicates that the prediction was incorrect. For a good-quality classifier model, TP and TN must be high, while FP and FN must be low.

According to Figure 8, the best results were achieved with DT and RF when trained with features selected by GA. High false alarms were obtained with SVM and kNN models, especially when training the SVM algorithm with features selected by the CFS algorithm (616). A false negative refers to 225; an actual value is positive, but the model predicted negative; the worst value was obtained with the SVM model (11,511). For the TP, when the actual value is positive and the predicted value is also positive, the best values were achieved with DT and RF when trained with GA features (59,391). For the TN, the best results (400,703) were obtained with DT when trained with both CFS and GA features, and also when trained with RF models with GA and SVM with CFS. According to these results, the highest confusion matrix scores were obtained when DT and RF classifiers were trained using GA-selected features.

**Figure 9:**
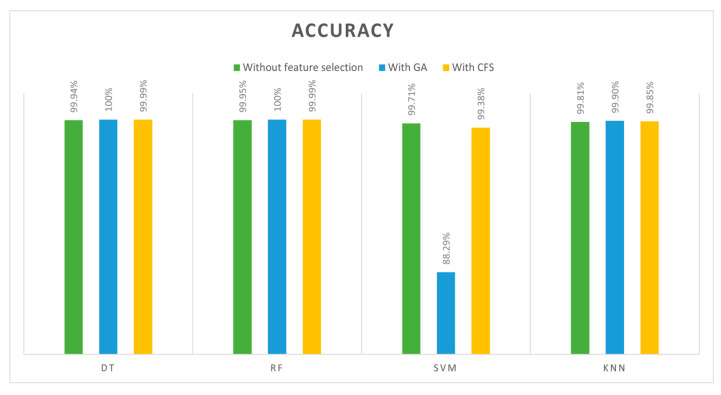
Accuracy results.

To quantify and compare the quality of the models, we calculated different measures from the confusion matrix, which include accuracy, precision, recall, and F1 score. The first measure is accuracy, where the accuracy of a model is a measure of how often it is correct. Table 12 compares the accuracy achieved across four classifiers (DT, SVM, RF, and kNN) and three features’ cases (trained with all features, trained with GA-selected features, and trained with CFS-selected features). A clear comparison can be seen in Figure 9. When trained with features selected by GA, the DT and RF algorithms showed the best results with 100% accuracy. However, the SVM model with GA features achieved less accuracy (88.2922%); this occurred because SVMs do not work well with large datasets with strong correlations between features, in contrast to DT and RF classifiers.

**Figure 10:**
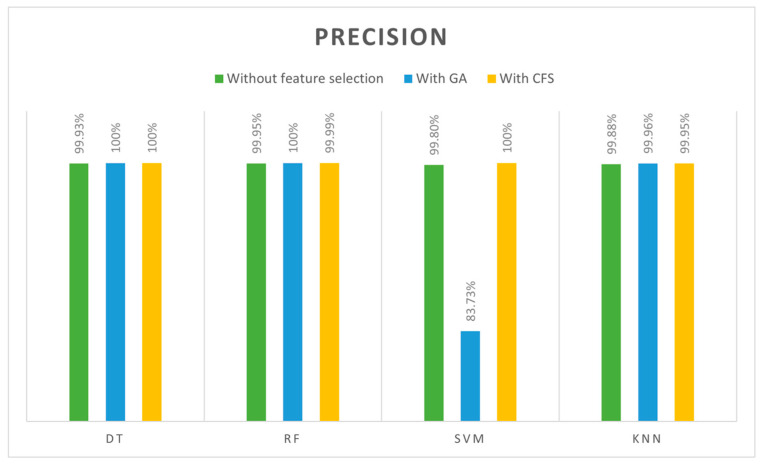
Precision results.

Precision is another metric used to identify the quality of models, namely how many intrusions a model predicts with a lower FP. In Table 13, precision is shown for four classifiers (DT, SVM, RF, and kNN) with three feature cases (all features, GA-selected features and CFS-selected features). Figure 10 compares classifiers in terms of precision. Precision results were outperformed for DT models trained with GA and CFS features, RF models trained with GA-selected features and SVM models trained with GA (100%). However, the SVM model with GA features achieved the lowest precision (83.7342%), and SVM is not robust with a large dataset, which caused this.

**Figure 11:**
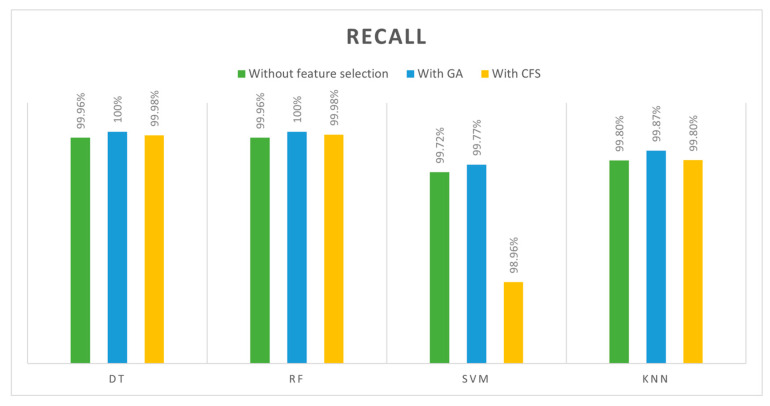
Recall results.

The third metric chosen to evaluate models is recall. The recall value is the ratio of attacks that were expected to those that actually occurred, so a high R-value is needed to determine what proportion of attacks were actually detected. In Table 14, recall is shown for four classifiers (DT, SVM, RF, and kNN) with three feature cases (all features, GA-selected features, and CFS-selected features). Figure 11 compares classifiers in terms of recall. The best recall results were obtained by training DT and RF with features selected by GA (100%). In contrast, when SVM classifiers were trained with features selected using CFS algorithms, it produced the worst recall result (98.9628%). With the results of DT and RF classifiers, using them in IDS makes sense.

**Figure 12:**
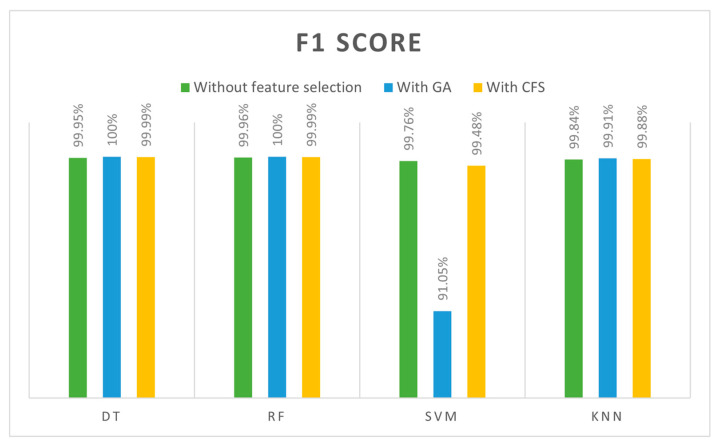
F1 score results.

The last metric is the F1 score; it combines precision and recall, providing an overall score. In Table 15, F1 score results are presented for four classifiers (DT, SVM, RF, and kNN) in three feature cases (all features, GA-selected features, and CFS-selected features). Figure 12 compares the classifiers in terms of the F1 score. DT and RF achieved the best F1 scores (100%) when they were trained with GA-selected features. By contrast, SVM was trained with GA features, resulting in the lowest F1 score. Based on the given results of the SVM classifier, it is not ideal for IDS due to its difficulty in dealing with large datasets and its sensitivity to kernel type.

According to the overall comparisons of 12 models, the best accuracy, precision, recall, and F1 scores were achieved by both DT and RF classifiers when they trained with GA features (13 features) with 100% across all metrics. Based on training and testing time, we found that DT outperformed the RF with (0.0644 s) training for DT and (0.7264 s) for the RF. Consequently, the best model was achieved when GA was used to select the features and DT was used as the classifier algorithm. Due to the high-performance results, they were achieved with our selected dataset to detect DoS attacks in IoT networks. Furthermore, the GA finds features quickly and accurately and works simply. In terms of the DT classifier, it is simple, fast, and efficient. The lowest results, however, were obtained with the SVM algorithm, especially when GA selected the features. This occurred as a result of the SVM algorithm’s inability to cope with large datasets that have strong correlations between features. We were able to achieve 100% accuracy values in some cases; however, these results were obtained when the models were trained with a specific dataset and under specific conditions. We understand the need for comprehensive validation and testing in real IoT environments and different datasets to ensure the generalizability and robustness of our proposed model as part of our future work.

Compared to other machine learning-based security solutions for IoT networks, our work provides more information about using anomaly IDS in IoT networks. Further, many previous studies used outdated datasets, lacked IoT traces, lacked modern attack types, and hardly contained features related to IoT. In this paper, we compared available datasets for this purpose and selected the dataset that included the most recent traffic attacks collected in real-time and simulated attacks on IoT networks. Also, instead of training the classifier with all features in the dataset, we selected the most related features to detect different types of DoS attacks. Particularly for improving detection accuracy and training speed, we selected the most relevant and important features (13 out of 86 features). In order to select the most appropriate feature selection algorithms, we compared two well-known algorithms, which are GA and CFS, with four classifiers (DT, RF, SVM, and kNN). As well, we trained the classifiers with all dataset features in order to compare them. Furthermore, most of the previous studies did not consider time spent training and testing as an important measure for benchmarking classifiers. To evaluate the performance of different models, we used four metrics: accuracy, precision, recall, and F1 score, in addition to training and testing time.

## 7. Conclusions and Future Work

IoT systems are susceptible to a wide range of security attacks, and of those that prevent legitimate IoT network users from accessing their services, DoS is one of the most common. Therefore, to ensure that systems, devices, and data are secured and protected effectively, robust techniques and mechanisms to counter attacks against IoT networks are needed. To address these issues and prevent hackers from attacking IoT networks successfully by using DoS attacks against devices, this study aimed to propose a hybrid defense mechanism based on anomaly detection, IDS, and ML techniques. In this study, we compared and selected the optimal dataset for training the system to detect DoS attacks in the IoT network. Then, we compared two feature selection algorithms to select the relevant and most important features. In order to obtain the best performance for our IDS, we used the most well-known supervised ML algorithms: KNN, DT, RF, and SVM. Each of these selected algorithms were trained in three ways: first, using all the features (without applying any feature selection techniques), and then using features selected by GA. Finally, we trained it with features selected by CFS. Additionally, we evaluated and compared the algorithm’s performance using a confusion matrix. We found that the best performances are obtained when training the DT and RF using the features selected by GA with 100% across the four metrics.

For future work, we will try selecting fewer features and experimenting with other feature selection algorithms. We will also test our system by downloading the selected model to the Raspberry Pi microcontroller board. Moreover, we will conduct experiments and evaluate our proposed model’s performance using other datasets, such as the new dataset CIPMAIDS2023-1, or build our own dataset containing new attack data collected from real-world IoT environments and implement other types of classifier algorithms, such as deep learning.

## Figures and Tables

**Table 1:** Analysis of related work.

Author	Year of Publication	Detection Model	Targeted Environment	Attacks Identified
Aversano et al. [1]	2021	DNN	IoT Systems	Scanning, TCP DoS, UDP DoS, TCP DDoS, UDP DDoS, HTTP DoS, Mirai, and Xbash
Verma et al. [3]	2020	RF, ad boost, gradient boosted machines, extremely randomized trees, classification and regression trees, and multi-layer perceptron	IoT Applications	DoS attack
Khatib et al. [6]	2021	LDA, SVM, KSVM, Nystroms, DT, RF, and LRand adaboost	IoT Networks	Fuzzers, Analysis, Backdoors, DoS, Exploits, Generic, Reconnaissance, Shellcode, and Worms
Naung Soe et al. [8]	2020	SVM	IoT Networks	Fuzzers, Analysis, Backdoors, DoS, Exploits, Generic, Reconnaissance, Shellcode, and Worms
Khammassi et al. [12]	2017	LR, GA, DT	IoT system	Attack types included in the KDD99 and UNSW-NB15 datasets
Mukherjee et al. [13]	2022	LR, Naïve Bayes (NB), DT, RF, ANN	IoT Networks	DoS, Data Type Probing (DTP), Malicious Control (MC), Malicious operation (MO), Scan, Spying, and Wrong Setup (WS)
Brun et al. [14]	2018	RNN	IoT Networks	UDP flood, TCP SYN, sleep deprivation, barrage, and broadcast
Tyagi et al. [15]	2021	KNN, LR, SVM, MLP, DT, and RF	IoT Networks	DDoS, DoS, reconnaissance, and information theft
Thamilarasu et al. [16]	2019	Deep Neural Network (DNN)	IoT Networks	Blackhole, Opportunistic Service, DDoS, Sinkhole, and Wormhole
Anthi et al. [17]	2018	NB	IoT Networks	Network scanning, probing, and simple forms of DoS attacks
Ye et al. [18]	2018	SVM	Software-defined network	DDoS attack
Kostas [19]	2018	Naive Bayes, QDA, RF, ID3, AdaBoost, MLP, and KNN	Software-defined network	Bot, DDoS, DoS GoldenEye, DoS Hulk, DoS Slowhttptest, DoS slowlorFTPPatator, Heartbleed, Infiltration, PortSca, SSH-Patator, and Web Attack
Alsheikh et al. [20]	2014	J48, RF, and KNN	Medical wireless sensor networks	Not mentioned
Hasan et al. [21]	2019	LR, SVM, DT, RF, and ANN	IoT Sensors	DOS, Data Type Probing, Malicious Control, Malicious Operation, Scan, and Spying
Ramadan et al. [22]	2020	Light Convolutional Neural Network with Gated Recurrent Neural Network (LCNNGRNN) algorithm	IoT Networks	DoS, U2R, and R2L Probe
Lopez-Martin et al. [23]	2017	Conditional variational autoencoder	IoT Networks	DoS, U2R, and R2L Probe
Yihunie et al. [24]	2019	Stochastic Gradient Decent, RF, Logistic Regression, SVM, and Sequential Model	Not mentioned	DoS, U2R, and R2L Probe
Kim et al. [25]	2005	SVM	Not mentioned	DoS attack
Meng [26]	2011	Neural networks, SVM, and DT	Not mentioned	Probe, DoS, U2R, and R2L
Al-Janabi et al. [27]	2011	ANN	Wireless Networks	DoS, Probe, U2R, and R2L
Shurman et al. [28]	2019	A hybrid design of signature-based IDS and anomaly-based IDS	IoT Networks	DoS
Mamatha et al. [29]	2019	LSSVM	Not mentioned	Attack types included in the KDD99 and UNSW-NB15 datasets
Albulayhi et al. [30]	2022	Bagging, Multilayer Perception, J48, and IBk	IoT system	Mirai, DoS, scan, and MAS
Krishnan et al. [31]	2021	SVM, Random Forest, and XGBoos	IoT system	DoS and spoofing
Qaddoura et.al. [32]	2021	SVM Gradient Descent (SGD), LR, NB, SLFN, and oversampling	IoT system	DoS, Mirai, MITM, and scan
Choudhary et al. [33]	2021	DNN and SVM	IoT Networks	DDoS, DoS, and Replay
Mohan Sai et al. [34]	2021	SVM	IoT Networks	DoS attack

**Table 3:** Compare publicly available datasets.

Dataset Name	Year of Creation	Number of DoS Instances	Number of Features	Advantages	Disadvantages
UNSWNB15	2015	16,353	47	- The testing set does not contain any duplication - Some http connections and TCP/IP attributes are included in the content characteristics	- Training set with high-duplicated records - Has scarce data
KDD 99	1998	3,883,370	41	- Highest records for the DoS class	- Outdated data - Huge number of redundant records
NSL-KDD	2000	53,385	42	- Improved version of the KDD99 - The train set does not include redundant records - Prevents bias in the ML algorithm during training	Outdated data
IoTID20	2020	59,391	86	- It replicates a modern trend of IoT network communication - It contains various types of IoT attacks and families - Up-to-date dataset - Attack traffic is collected in real-time	The data types and formats of some features are not suitable for ML algorithms
Bot-IoT	2018	33,003,929	46	- Resolving the existing drawbacks of capturing network information in current datasets - Accurate labeling Diverse and recent attacks	- Does not reflect a realistic IoT network - Normal traffic is generated by botnet VMs - The attacks were not unique to IoT networks

**Table 4:** Normal and attacked instances in the IoTID20 dataset.

Binary Label Distribution	Instances
**Normal**	40,073
**Anomaly**	585,710

**Table 5:** IoTID20 anomaly data distribution.

Category Label Distribution	Instances
**Normal**	40,073
**DoS**	59,391
**Mirai**	415,677
**MITM**	35,377
**Scan**	75,265

**Table 6:** Advantages and disadvantages of GA and CFS algorithm.

Algorithm	Advantages	Disadvantages
GA	- A quick and accurate way to find a good quality solution - Easy to code	- No guaranteed optimal solution - Parameter selection is difficult
CFS	- Tests the predictive power of genes - Comparatively less computational complexity than GA - Overfitting is less likely than GA	- Due to the heavy reliance on the model, the data may not fit well

**Table 7:** Deleted features.

**Features Deleted**	‘Src IP’, ‘Flow ID’, ‘Sub Cat’, ‘Dst IP’, ‘Timestamp’, ‘Label’

**Table 8:** Encoding.

Category	Numerical Value
**DoS**	1
**Normal**	0

**Table 9:** GA features.

SI	Feature Name	Data Type	Description
1	Src _Port	Int64	Source Port Number
2	Dst _Port	Int64	Destination Port Number
3	Tot _Fwd_Pkts	Int64	Total packets in the forward direction
4	Tot _Bwd _Pkts	Int64	Total packets in the backward direction
5	Fwd_ Pkt_ Len _Max	Float64	The maximum size of the packet in the forward direction
6	Flow_IAT _Mean	Float64	Mean time between two packets sent in the forward direction
7	Bwd_IAT_Std	Float64	Standard deviation time between two packets sent in the backward direction
8	Bwd_URG_Flags	Int64	Number of times the URG flag was set in packets travelling in the backward direction (0 for UDP)
9	SYN_Flag_Cnt	Int64	Number of packets with SYN
10	URG_Flag_Cnt	Int64	Number of packets with URG
11	Down/Up_Ratio	Float64	Download and upload ratio
12	Idle_Mean	Float64	Mean time a flow was idle before becoming active
13	Idle Max	Float64	The maximum time a flow was idle before becoming active

**Table 10:** CFS features.

SI	Feature Name	Data Type	Description
1	Idle_Min	Float64	The minimum time a flow was idle before becoming active
2	Bwd_Header_Len	Int64	Total bytes used for headers in the backward direction
3	Fwd _IAT_Mean	Float64	Mean time between two packets sent in the flow
4	TotLen_Fwd_Pkts	Int64	The total size of the packet in forward direction
5	Fwd_ Pkt_ Len _Max	Float64	The maximum size of the packet in forward direction
6	Fwd Pkts/s	Float64	Number of forward packets per second
7	Flow_IAT _Std	Float64	Number of forward packets per second
8	Pkt_Len_Var	Float64	Variance length of a packet
9	Idle_Std	Float64	Standard deviation time a flow was idle before becoming active
10	Fwd_IAT_Max	Float64	The maximum time between two packets sent in the forward direction
11	Pkt_Len_Max	Float64	The maximum length of a packet
12	Flow_IAT_Min	Float64	The minimum time between two packets sent in the flow
13	Flow_IAT_Mean	Float64	Mean time between two packets sent in the forward direction

**Table 11:** Training and testing times.

Classifier	Training Time (s).			Testing Time (s).		
	All Features	GA	CFS	All Features	GA	CFS
**DT**	0.1718	0.0644	0.0743	0.0626	0.0099	0.0637
**RF**	1.0024	0.7264	0.8992	0.0700	0.1862	0.2008
**SVM**	4.1583	5.2146	45.0077	1.5224	5.8803	63.2915
**kNN**	0.01241	0.2128	0.1597	2.7450	7.2853	6.7626

**Table 12:** Accuracy results.

Classifier	Without Feature Selection	With GA	With CFS
**DT**	99.936%	100%	99.9859%
**RF**	99.9482%	100%	99.9859%
**SVM**	99.7106%	88.2922%	99.3807%
**kNN**	99.8081%	99.8964%	99.8512%

**Table 13:** Precision results.

Classifier	Without Feature Selection	With GA	With CFS
**DT**	99.9341%	100%	100%
**RF**	99.9544%	100%	99.9949%
**SVM**	99.797%	83.7342%	100%
**kNN**	99.8782%	99.9562%	99.946%

**Table 14:** Recall results.

Classifier	Without Feature Selection	With GA	With CFS
**DT**	99.9594%	100%	99.9764%
**RF**	99.9594%	100%	99.9815%
**SVM**	99.7211%	99.7744%	98.9628%
**kNN**	99.8022%	99.8704%	99.8047%

**Table 15:** F1 score results.

Classifier	Without Feature Selection	With GA	With CFS
**DT**	99.9594%	100%	99.9764%
**RF**	99.9594%	100%	99.9815%
**SVM**	99.7211%	99.7744%	98.9628%
**kNN**	99.8022%	99.8704%	99.8047%

## Data Availability

Data are contained within the article.
